# Automated hippocampal volume measurement: agreement analysis between HIPS and volBrain software

**DOI:** 10.1590/0100-3984.2025.0003

**Published:** 2025-08-25

**Authors:** Federico Biafore, Jorge Docampo, Germán Duca

**Affiliations:** 1 Fundación Científica del Sur, Lomas de Zamora, Provincia de Buenos Aires, Argentina.; 2 Tomografía Computada Buenos Aires Centro de Diagnóstico (TCba), Ciudad Autónoma de Buenos Aires, Argentina.

**Keywords:** Hippocampus, Magnetic resonance imaging, Alzheimer disease, Image processing, computer-assisted, Reproducibility of results, Hipocampo, Ressonância magnética, Doença de Alzheimer, Processamento de imagem assistida por computador, Reprodutibilidade dos testes

## Abstract

**Objective:**

To perform an agreement analysis between volBrain and HIPS software for measuring hippocampal volume and its associated asymmetry index.

**Materials and Methods:**

We evaluated volumetric T1-weighted magnetic resonance imaging scans from radiologically normal subjects (n = 50; age range, 25–75 years). Correlation and Bland-Altman plots were generated. The Pearson correlation coefficient (*r*) and the intraclass correlation coefficient of absolute agreement between volBrain and HIPS software were calculated for each measurement.

**Results:**

For each hippocampus and its combined volume, a very high correlation was found between the methods (*r* ≥ 0.96 for absolute values and *r* ≥ 0.93 for relative values), along with a systematic bias (primarily additive). Consistently, HIPS (with the Kulaga-Yoskovitz protocol) reported smaller volumes than did volBrain. The average difference ranged from 8.2% to 9.1% for absolute values and from 7.9% to 8.7% for relative values. The asymmetry index exhibited a strong correlation (*r* = 0.82) with no significant bias, although 14% of cases showed opposite signs. The average asymmetry index difference was 32.7%. The intraclass correlation coefficient of absolute agreement ranged from 0.61 to 0.83, reflecting moderate to good agreement overall.

**Conclusion:**

Our results indicate that the two methods are not interchangeable for evaluating hippocampal volume and its associated asymmetry index.

## Abstract (pt)

**Objetivo:**

Realizar uma análise de concordância entre os *softwares* volBrain e HIPS para medir o volume do hipocampo e seu índice de assimetria associado.

**Materiais e Métodos:**

Foram usadas imagens volumétricas de ressonância magnética ponderadas em T1 de 50 indivíduos radiologicamente normais (25–75 anos). Foram realizados gráficos de correlação e de Bland-Altman. Os coeficientes de correlação de Pearson (*r*) e o coeficiente de correlação intraclasse de concordância absoluta entre os dois métodos de medição foram calculados em cada caso.

**Resultados:**

Os volumes do hipocampo, isolados e combinados, apresentaram correlação extremamente alta entre os métodos (*r* ≥ 0,96 para valores absolutos e *r* ≥ 0,93 para valores relativos), juntamente com um viés sistemático predominantemente aditivo. Os volumes medidos pelo HIPS (protocolo de Kulaga) foram consistentemente menores que os do volBrain, com diferença média entre os métodos de 8,2% a 9,1% para valores absolutos e de 7,9% a 8,7% para valores relativos. Para o índice de assimetria, houve forte correlação (*r* = 0,82) sem viés significativo, embora 14% da amostra apresentassem sinais opostos. A diferença média entre os métodos foi de 32,7%. Os valores de correlação intraclasse de concordância absoluta variaram de 0,61 a 0,83, refletindo uma concordância geral de moderada a boa.

**Conclusão:**

Os resultados sugerem que os dois métodos não são intercambiáveis para avaliar o volume do hipocampo e seu índice de assimetria associado.

## INTRODUCTION

Structural changes in the brain are often associated with various neurodegenerative or psychiatric conditions, as well as with normal aging. These changes can alter the properties of images acquired, such as intensity values on magnetic resonance imaging (MRI) and computed tomography, and may also lead to morphological variations, including changes in the volume of different brain tissues and structures^(1-3)^. The high spatial resolution of three-dimensional (3D) images from clinical high-field (1.5-T and 3.0-T) MRI systems, combined with advances in segmentation and quantification algorithms, has driven rapid growth in this field of research, significantly impacting clinical practice, especially over the last decade. As a result, volumetric MRI analysis has demonstrated significant potential as a tool for the diagnosis and monitoring of various neurological diseases^(4-9)^.

Hippocampal volumetry is widely used for studying and monitoring diseases such as temporal lobe epilepsy, Alzheimer’s disease, and schizophrenia. The value of these measurements as imaging biomarkers, along with related metrics like the asymmetry index, has been supported by extensive research^(10-14)^. Although manual volumetric techniques performed by specialists are considered the gold standard, their time-consuming nature makes them impractical for routine clinical use, and achieving consistent reproducibility remains challenging. As a result, there is a growing use of automated techniques based on probability atlases, which operate without user intervention. These methods incorporate spatial information, in addition to signal intensity at the pixel or voxel level, to classify tissues and structures^(1)^. These atlases are created from MRI studies of cohorts of healthy subjects that have been spatially aligned and intensity-normalized, ensuring a shared geometric and intensity domain across the dataset. Including a large number of subjects accounts for anatomical variability across individuals^(1,5)^. From such atlases, segmentations and volumetric calculations can be performed automatically at different scales for a given case (once the images have been normalized to the atlas space), from macroscopic tissues to subcortical structures^(1,5,7)^. Several free, commercially licensed software packages are currently available to perform this task^(15-23)^. One of these is the volBrain platform (www.volbrain.org), which offers a suite of MRI volumetric analysis tools. It requires no software installation or operation by the user; the image to be analyzed is simply uploaded to the website. The platform handles image preprocessing and processing, delivering a report with the corresponding volumetric measurements in about 20 min. Among the tools available on the platform, the volBrain module performs brain segmentation and volumetry at multiple scales from a 3D T1-weighted MRI scan, with a recommended 1-mm isotropic resolution in Neuroimaging Informatics Technology Initiative (NIfTI) format.

The volBrain report includes measurements of the total intracranial volume (TIV), total gray matter, white matter, cerebrospinal fluid, lateral ventricles, cerebellum (left and right), and volumes of subcortical structures such as the putamen, caudate, globus pallidus, thalamus, hippocampus, amygdala, and accumbens^(19)^.

In contrast, the HIPS module is specifically designed for hippocampal volumetry, including the parcellation of the hippocampus into its substructures. This module can work with only one 3D T1-weighted (monomodal) MRI scan or by adding a 3D T2-weighted (bimodal) image^(20)^.

The aim of this study was to perform an agreement analysis between the two modules for measuring the volume of the hippocampus and the corresponding asymmetry index using T1-weighted MRI scans from radiologically normal subjects.

## MATERIALS AND METHODS

For this retrospective analysis, we used volumetric T1-weighted MRI scans from radiologically normal adults (n = 50; age range, 25–75 years) without a significant medical history. All images were acquired in 3.0-T scanners.

One of the image sets (group 1) was obtained from our institution and consists of images of patients with a history of headaches (n = 20; 10 males and 10 females; age range, 25–40 years), acquired in 3D fast spoiled gradient-echo sequences with a resolution of 1 × 1 × 1.2 mm^3^. All of the patients gave written informed consent before undergoing the imaging scans.

The second set of images (group 2) consists of images of cognitively normal adults (n = 30; 15 males and 15 females; age range, 43–75 years), sourced from the Open Access Series of Imaging Studies database^(24)^. The images were acquired in 3D fast spoiled gradient-echo and 3D magnetization-prepared rapid gradient-echo sequences with resolutions of 1 × 1 × 1.2 mm^3^ and 1.2 × 1.05 × 1.05 mm^3^, respectively.

Both image sets were evaluated by a senior neuroradiologist and showed no abnormalities. Our multicentric population sample covered a wide age range, and the images were acquired in different 3D sequences commonly used in routine practice.

### Image processing

The images were anonymized and converted to NIfTI format before being processed on the volBrain platform.

*Preprocessing* – Both modules perform an image preprocessing pipeline, which includes the application of a noise removal filter, correction for field inhomogeneity, normalization to Montreal Neurological Institute space, intensity normalization, and extraction of the intracranial cavity. The detailed description can be found in previous works^(19,20)^.

*volBrain –* All segmentations, except for that of the hippocampi, are based on adaptations of probabilistic atlases and manually segmented libraries. In the specific case of the hippocampus, volBrain follows the Alzheimer’s Disease Neuroimaging Initiative harmonized protocol, which defines procedures to standardize hippocampal segmentation. The protocol was designed by the European Alzheimer’s Disease Consortium to establish consensus on hippocampal segmentation for clinical and research applications, while also serving to validate automated segmentation algorithms^(19,25)^.

*HIPS* – We selected the Kulaga-Yoskovitz monomodal segmentation protocol, which divides the hippocampus into three substructures^(20)^: CA1-3, CA4/DG, and the subiculum. The total hippocampal volume is obtained by summing the volumes of those substructures, as detailed in the corresponding report. In addition, both modules generate segmentation masks of the structures in NIfTI format, which can be merged with the MRI images for evaluation purposes.

The reports include the absolute and relative values (expressed as a percentage of the TIV) for the segmented structures, along with reference ranges of normal values. From those, the absolute and relative values were obtained. The asymmetry index was calculated as follows:

$$AI=100\frac{\left(H_{R}-H_{L}\right)}{\left[\frac{\left(H_{R}+H_{L}\right)}{2}\right]}$$

where *AI* is the asymmetry index and where *H_R_*, *H_L_*, and *H_R_* + *H_L_* are the absolute values for the volumes of the right hippocampus, left hippocampus, and total hippocampus, respectively.

### Statistical analysis

Correlation and Bland-Altman scatter plots were generated for each measurement. The Pearson correlation coefficient (*r*) and absolute intraclass correlation coefficient (ICC) were computed to assess the degree of linear association and agreement between the two measurement methods^(26,27)^, respectively. Because the absolute ICC considers any difference between measurements as discordance, is a useful statistical tool to assess whether the two methods are interchangeable.

The Shapiro-Wilk normality test was conducted for each variable and its corresponding differences to ensure the proper application of statistical tools. The analysis was performed with the Excel-based XLSTAT package (Addinsoft, New York, NY, USA).

## RESULTS

**Figure 1:**
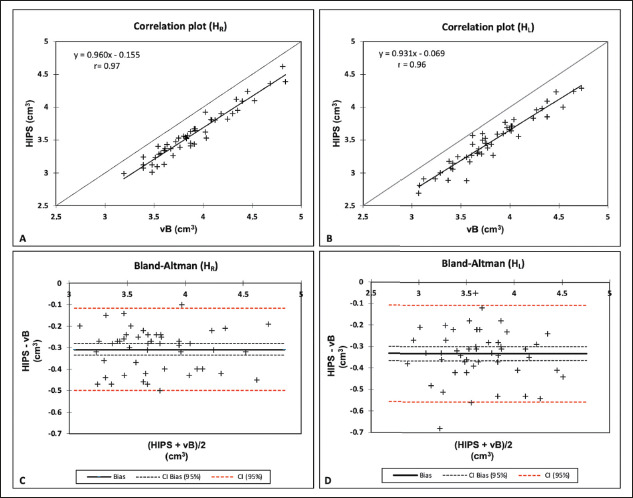
Correlation and Bland-Altman plots for the volumes of the right hippocampus (A,C) and left hippocampus (B,D). H_R_, right hippocampus; H_L_, left hippocampus; vB, volBrain.

**Figure 2:**
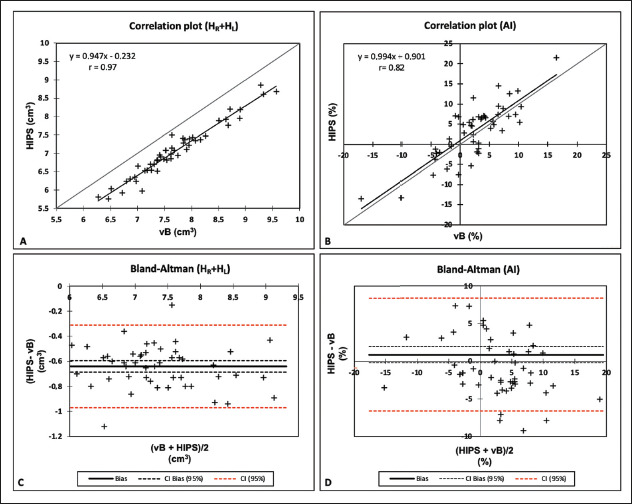
Correlation and Bland-Altman plots for total hippocampal volume (A,C) and the asymmetry index (B,D). H_R_, right hippocampus; H_L_, left hippocampus; AI, asymmetry index; vB, volBrain.

**Figure 3:**
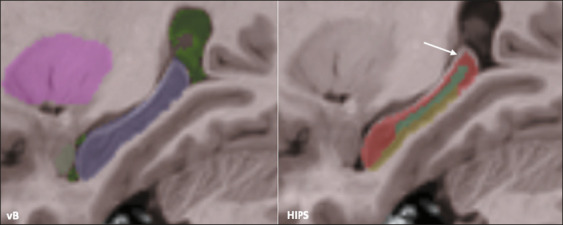
Example of hippocampal segmentation with volBrain (left) and HIPS (right). The arrow highlights regions excluded from the hippocampal segmentation by the HIPS method.

The mean values for the two methods and the agreement analysis parameters are presented in Tables 1 and 2, respectively. Figures 1 and 2 display the results of the correlation and Bland-Altman analyses for absolute values and the asymmetry index. The two methods showed very high linear correlations for the volumes of the right hippocampus, left hippocampus, and total hippocampus, for absolute (*r* ≥ 0.96) and relative values (*r* ≥ 0.93), as indicated in Table 2. As illustrated in Figures 1A, 1B, and 2A, respectively, the data for those three variables lie below the identity function (diagonal line), indicating that HIPS module measurements are consistently lower than are those from the volBrain module. This is demonstrated by the Bland-Altman plots in Figures 1C, and 1D. Likewise, Table 2 shows that the mean differences (biases) for absolute and relative values are less than zero, with their respective confidence intervals excluding the zero line (equality between measurements). The results clearly indicate that there is a systematic bias between the two modules for those three variables. Using the mean of both methods as the best estimate of the measurement, the bias can be expressed as a percentage of the mean for each estimator (bias%, Table 2). The difference between the two methods ranges, on average, from 8.2% to 9.1% for absolute values and from 7.9% to 8.7% for relative values. Figure 3 shows an example of segmentation differences between the two methods for the right hippocampus of the same subject.

**Table 1:** Mean values and standard deviations.

Variable	Absolute values		Relative values	
	HIPS Mean ± SD	volBrain Mean ± SD	HIPS Mean ± SD	volBrain Mean ± SD
Right hippocampal volume (cm^3^)	3.59 ± 0.38	3.89 ± 0.38	0.257 ± 0.019	0.278 ± 0.021
Left hippocampal volume (cm^3^)	3.48 ± 0.39	3.81 ± 0.40	0.249 ± 0.023	0.272 ± 0.024
Total hippocampal volume (cm^3^)	8.85 ± 0.73	9.57 ± 0.75	0,506 ± 0.040	0.550 ± 0.043
Asymmetry index (%)	3.17 ± 6.78	2.28 ± 5.3	*	*

Relative volumetric values are expressed as a percentage of the TIV.

* Asymmetry index values are identical for absolute and TIV-normalized volumes.

**Table 2:** Results of the agreement analysis.

Variable	Absolute values HIPS vs. volBrain				Relative values HIPS vs. volBrain			
	r*	ICC_A_	Bias [95% CI]	Bias%	r*	ICC_A_	Bias [95% CI]	Bias%
Right hippocampal volume (cm^3^)	0.97	0.73	‒0.308 [‒0.335; ‒0.280]	‒8.2%	0.93	0.61	‒0.021 [‒0.023; ‒0.019]	‒7.9%
Left hippocampal volume (cm^3^)	0.96	0.71	‒0.332 [‒0.364; ‒0.299]	‒9.1%	0.94	0.64	‒0.023 [‒0.025; ‒0.020]	‒8.7%
Total hippocampal volume (cm^3^)	0.97	0.71	‒0.640 [‒0.688; ‒0.593]	‒8.6%	0.95	0.61	‒0.044 [‒0.047; ‒0.040]	‒8.3%
Asymmetry index (%)	0.83	0.81	0.89 [‒0.19; 1.97]	32.7%	†	†	†	†

* *P* < 0.0001.

† Asymmetry index values are identical for absolute and relative values. ICC_A_, absolute ICC; Bias, mean difference between the methods; Bias%: bias expressed as a percentage of the mean value of the estimator of the measure; CI, confidence interval

For the asymmetry index (Figure 2B), the correlation between the two methods was strong (*r* = 0.82), albeit slightly weaker than that for the other three measurements. No significant bias was observed in the data distribution. The graph also shows that in seven cases (14% of the sample), the asymmetry indices had opposite signs. The Bland-Altman plot (Figure 2D) and Table 2 show that the bias is close to the zero line (0.89), with the confidence interval encompassing this value. In terms of bias (%), the difference between the two methods is, on average, 32.7%.

The absolute ICC values for absolute and relative measurements ranged from 0.71 to 0.73 and from 0.61 to 0.64, respectively, whereas the asymmetry index was 0.81. According to the criteria established by Koo et al.^(26)^, the reliability was classified as moderate for the right hippocampus, left hippocampus, and total hippocampus, whereas it was classified as good for the asymmetry index.

## DISCUSSION

The agreement analysis between HIPS and volBrain revealed a primarily additive systematic bias when measuring the absolute and relative volumes of each hippocampus and their combined volume in a radiologically normal population over a wide age range. Although those measurements exhibited a very strong linear correlation, the absolute ICC indicated only a moderate level of absolute agreement. In general, the absolute ICC values were lower for the relative volumetric measurements than for the absolute ones, because of greater data dispersion of the TIV estimates in both methods.

Although the statistical analysis defines confidence limits for agreement between the two methods, the acceptance of those limits should ultimately be guided by biological and clinical criteria relevant to the specific application of these measurements. Available evidence indicates that the average annual rate of hippocampal volume loss is approximately −0.8% in normal aging, −2.6% in mild cognitive impairment, and −4.4% in Alzheimer’s disease^(28)^. Given that the difference between the methods, based on the best estimates defined above, averages at least 7.9%, switching between HIPS and volBrain—particularly in a longitudinal study—could significantly impact the results and their interpretation.

When calculating the asymmetry index, we observed no systematic bias. It also achieved the highest absolute ICC value among all variables analyzed, although it did not meet the threshold for excellent reliability based on the Koo et al. criteria^(26)^. The average difference between the two methods was 32.7%. In addition, the indices showed an opposite sign in 14% of cases. These results also suggest that switching modules might have an impact on conclusions regarding the quantification of the asymmetry index. The overall results suggest that the two methods are not directly interchangeable for volumetric assessment of hippocampal structures and for determining the asymmetry index, unless the linear equation linking them is considered.

Selecting the most appropriate algorithm for MRI brain volumetry remains challenging because there is no gold standard for automated techniques. Variations in image processing techniques, segmentation methods, and anatomical definitions can result in substantial discrepancies between or among approaches. Previous studies have shown high variability in absolute hippocampal volume measurements across several automated methods, with differences ranging from 2.3% to 48.7%^(29,30)^. For TIV-normalized volumes, differences greater than 24% can be derived from previous works^(13)^. Given these results, the two methods evaluated in this study exhibit differences that can be classified as modest. However, these differences may be greater than those caused by the condition being studied.

The tools examined in this study, provided on a single platform, enable rapid generation of detailed volumetric reports. However, it is essential to critically assess the accuracy of the segmentations generated by the chosen software. Similarly, maintaining consistency across acquisition, processing, and segmentation methods is crucial to prevent errors and biases that could compromise clinical decisions.

Several limitations of this study must be considered. First, the study sample included only subjects without pathological hippocampal alterations (e.g., severe atrophy), which may restrict the generalizability of the findings to clinical populations. In addition, the population sample was not stratified based on the MRI scanner model or manufacturer to assess significant differences among them, because such stratification would have resulted in subgroups with insufficient sample sizes for robust statistical analysis. Finally, the study did not assess whether differences in hippocampal segmentation between the two methods occur systematically in specific anatomical regions, leaving potential spatial biases unaddressed.

## CONCLUSION

The agreement analysis performed suggests that the volBrain and HIPS modules cannot be considered interchangeable for the volumetric assessment of the hippocampi and the associated asymmetry index.
